# The Effect of Estradiol and Progesterone on *Toll Like Receptor* Gene Expression in A Human Fallopian Tube Epithelial Cell Line

**DOI:** 10.22074/cellj.2016.3840

**Published:** 2016-01-17

**Authors:** Zahra Zandieh, Fatemehsadat Amjadi, Mahnaz Ashrafi, Abbas Aflatoonian, Alireza Fazeli, Reza Aflatoonian

**Affiliations:** 1 Department of Anatomy, School of Medicine, Iran University of Medical Sciences, Tehran, Iran; 2 Department of Obstetrics and Gynecology, School of Medicine, Iran University of Medical Sciences, Tehran, Iran; 3 Research and Clinical Center for Infertility, Shahid Sadoughi University of Medical Sciences, Yazd, Iran; 4 Academic Unit of Reproductive and Developmental Medicine, The University of Sheffield, Sheffield, United Kingdom; 5 Department of Endocrinology and Female Infertility, Reproductive Biomedicine Research Center, Royan Institute for Reproductive Biomedicine, ACECR, Tehran, Iran

**Keywords:** Estradiol, Progesterone, Fallopian Tube, Toll Like Receptors

## Abstract

**Objective:**

Toll like receptors (*TLRs*) are one of the main components of the innate im- mune system. It has been reported that expression of these receptors are altered in the female reproductive tract (FRT) during menstrual cycle. Here we used a fallopian tube epithelial cell line (OE-E6/E7) to evaluate the effect of two sex hormones in modulating *TLR* expression.

**Materials and Methods:**

In this experimental study, initially *TLR* gene expression in OE- E6/E7 cells was evaluated and compared with that of fallopian tube tissue using quanti- tative real time-polymerase chain reaction (qRT-PCR) and immunostaining. Thereafter, OE-E6/E7 cells were cultured with different concentrations of estradiol and progesterone, and combination of both. qRT-PCR was performed to reveal any changes in expression of *TLR* genes as a result of hormonal treatment.

**Results:**

*TLR1-10* genes were expressed in human fallopian tube tissue. *TLR1-6* genes and their respective proteins were expressed in the OE-E6/E7 cell line. Although estradiol and progesterone separately had no significant effect on *TLR* expression, their combined treatment altered the expression of *TLRs* in this cell line. Also, the pattern of *TLR* expres- sion in preovulation (P), mensturation (M) and window of implantation (W) were the same for all *TLRs* with no significant differences between P, M and W groups.

**Conclusion:**

These data show the significant involvement of the combination of es- tradiol and progesterone in modulation of *TLR* gene expression in this human fal- lopian tube cell line. Further experiments may reveal the regulatory mechanism and signalling pathway behind the effect of sex hormones in modulating *TLRs* in the hu- man FRT.

## Introduction

Infection within the upper regions of the female reproductive tract (FRT), particularly the fallopian tubes, can have serious consequences such as chronic pelvic inflammation, infertility and pregnancy complications (1, 2). For example, sexually transmitted diseases (STDs) that infect the upper regions of the FRT are a major worldwide health problem (3, 4). Approximately 8% of females annually develop pelvic inflammatory disease (PID) and after re-infection, this risk increases by 40-70% (5) . It is thus of paramount importance that infectious agents are quickly recognised and removed from the upper parts of the FRT. Characterization of the defense systems present within the FRT will assist the development of effective therapies or vaccination strategies against STDs.

The innate immune system is the first line of defense against infection. This system is able to identify what is foreign or non-self and produces adequate responses that lead to the pathogens being suppressed. Toll like receptors (*TLRs*) are a family of pattern recognition receptors that recognise pathogen-associated molecular patterns (PAMP) and constitute a major part of the innate immune system (6, 7). Until now, eleven members of this receptor family have been discovered in humans. Of these, *TLR1–9* are conserved between human and mouse. Using various methods including ectopic expression of mammalian cDNA in cell lines, some of the activating ligands of *TLRs* have been discovered. Each individual *TLR* is known to detect molecules (ligands) from varying classes of microbial agents (8, 9). Some *TLRs* like *TLR2* and its associated receptors *TLR1* and *TLR6* mainly react against Gram-positive bacteria by detecting molecules from mycobacteria and Gram-positive bacteria (10-12). Some PAMP, like lipoteichoic acid (LTA), can be detected only by *TLR2* (11).

In contrast, *TLR1* associates with *TLR2* to recognize triacylated lipoproteins (13), whereas *TLR2* together with *TLR6* detects diacylated lipoproteins and peptidoglycans (14-16). *TLR4* recognises lipopolysaccharides (LPS) which are present in Gram-negative bacteria (17-19). *TLR5* recognises bacterial flagellin (20). Other *TLRs* mainly react against viruses. For example, *TLR3* recognises RNA from double stranded RNA viruses (21, 22). Also, *TLR7* and 8 recognise RNA from single stranded RNA viruses and antiviral compounds such as imidazoquinolines (16, 19, 23), whilst *TLR9* recognises unmethylated CpG DNA found richly in prokaryotic genomes and DNA viruses (19, 24). Like *TLR1* and *TLR6*, *TLR10* is another *TLR2*-associated receptor and also highly homologous to *TLR2*, however, the function of this *TLR* is not completely understood (25-27). In addition, it was revealed that the *TLR* pathway has physiological relevance to human fertility *TLR* by playing roles in ovulation, sperm capacitation, fertilization and pregnancy (28-31). They were also shown to play a role in the pathophysiology of relevant disorders including endometriosis and poor ovarian response (32, 33).

Several studies have investigated the presence and the role of *TLRs* in the male and FRTs (34-43). Furthermore, the expression of *TLRs* in endometrial cell lines have been shown by Abussahoud et al. (44). It is evident that sex hormones modulate cells and their immune response potential, but varies throughout the FRT (45). Reports by us and others have demonstrated the existence of *TLRs* in FRT and the cycle-dependent expression of *TLRs* in the endometrium (34, 46). The effect of estradiol has also been showed in endometrial cells in several studies (47-49).

The cycle-dependent expression of *TLRs* in FRT implicates a role for sex hormones in regulation of *TLR* function in FRT. Here we used a fallopian tube epithelial cell line (OE-E6/E7) (50) to investigate the role of two sex hormones (estradiol and progesterone) in modulating *TLR* expression in the fallopian tube. This cell line has characteristics of human fallopian tube epithelial cells, including morphology, receptor expression and hormonal responses (50). Thereafter, the effect of the sex hormones and their combination as well as their antagonists on expression of *TLR1-10* in fallopian tube cells was investigated. This was done by testing *TLR* whether *TLR* expression is altered in the presence of sex hormones.

## Materials and Methods

### Fallopian tube tissue collection

This investigation was an experimental study approved by the Royan Institute Ethics Committee. Informed written consent was obtained prior to the collection of tissue samples. Human fallopian tube tissues were collected from 9 patients undergoing total abdominal hysterectomy for benign gynaecological conditions. The mean age of the women taking part in the study was 42 (range of 33-56) years with all in the secretory phase of their menstrual cycle. For genomic studies, fallopian tube tissue samples were immediately placed in RNAlater (Ambion, UK) and stored for 24 hours at 4˚C followed by immersion and storage in liquid nitrogen until the time of processing.

### Antibodies and peptides

Antibodies and peptides used in the experiments were obtained from Santa Cruz Biotechnology Inc. (USA). These were goat polyclonal antibodies specific for N-terminal domains of *TLR1, TLR2, TLR3, TLR5* and *TLR6*, and a goat polyclonal antibody specific for the C-terminal domain of *TLR4*. Blocking peptides specific for the respective antibodies were used to detect non-specific staining.

### Immunostaining

For immunostaining, OE-E6/E7 cells were cultured in four well chamber slides. They were cultured at 37˚C in Dulbecco’s Modified Eagle MediumF12 (DMEM-F12) culture medium (Invitrogen, UK) supplemented with 1% penicillin and streptomycin (Sigma-Aldrich, UK), 10% fetal calf serum (FCS, Invitrogen, USA) and L-glutamine (Invitrogen, USA) in 5% CO_2_ atmosphere. At confluency, the slides were washed five times with Ca^2+^ and Mg^2+^ free phosphate buffered saline (PBS, Gibco, USA), fixed with 5% formalin and stored at 4˚C until use.

Formalin-fixed slides were washed in PBS and then stained using a Vectastain Elite ABC peroxidase kit (Vector Laboratories Ltd, UK). In addition, to avoid non-specific binding, an avidin/ biotin blocking kit (Vector) was used. Briefly, slides were blocked for 1 hour at room temperature in PBS solution containing 0.2% v/v horse serum and 25% v/v avidin supplied in the blocking kit. The block was subsequently removed and slides were incubated for 2 hours at room temperature with primary antibody at an appropriate dilution using antibody diluent media (Dakocytomation Ltd, UK) and 250 ml biotin per ml of diluted antibody. Binding was then visualized by incubating the slide with peroxidase substrate 3-amino-9-ethylcarbazole (AEC) (Vector) for 10 minutes, washed in distilled water for 3 minutes and counterstained in 10% haematoxylin for 10 minutes. Slides were finally washed in tap water for 2 minutes and mounted with Aquamount (VWR International, UK).

Optimal staining was achieved by incubating slides with different concentrations of *TLR* antibodies (*TLR1, TLR2, TLR3, TLR4, TLR5, TLR6* with 4, 4, 10, 10, 4 and 10 μg/ml respectively). Negative control sections were obtained by blocking the primary antibody with the corresponding specific peptide. Immunostained sections were examined using an Olympus BH2 microscope (Olympus, UK).

### Toll like receptors expression in OE-E6/E7 cells

*TLR* expression in the OE-E6/E7 cell line was investigated and compared to fallopian tube tissue samples. OE-E6/E7 cells were cultured at 37˚C in DMEM (F12) supplemented with 1% penicillin and streptomycin, 10% FCS and L-glutamine in 5% CO_2_ atmosphere. At confluency, cells were washed with Ca^2+^ and Mg^2+^ free PBS and then harvested using trypsin-Ethylenediaminetetraacetic acid (EDTA, Invitrogen, USA) pelleted by centrifugation at 300 g for 5 minutes. One ml of TRIreagent (Sigma, UK) was added onto the pellet (5×106 cells). Thereafter, total RNA from pelleted cells was extracted following the standard protocol supplied by the manufacturer.

On the day of the experiment, fallopian tube tissues were removed from RNAlater and homogenised in 3 ml of TRI reagent using an Ultra-Turrax homogenizer for 2 minutes following the standard protocol supplied by the manufacturer.

Total RNA obtained from OE-E6/E7 cells and fallopian tube tissue samples (by using chloroform and isopropanol) were then treated with DNase I (DNA-free^TM^, Ambion, USA) to remove genomic DNA contamination from the samples. First strand cDNA was synthesized by reverse transcription using oligodT primers (Metabion, Germany) and SuperScript II (200 U/μl, Invitrogen, USA). RT controls were prepared without the enzyme (nonreverse- transcribed controls).

Polymerase chain reaction (PCR) was performed using prepared cDNA, Platinum Blue PCR Super Mix (Invitrogen, USA) and primers from Metabion (Table 1). The amplification was run for 40 cycles under the following conditions: initial heat at 95˚C for 30 secends, 59˚C to 65˚C for 30 seconds and final annealing at 72˚C for 30 seconds. All experiments included reverse transciptase (RT) controls as well as negative controls (no cDNA). PCR products were visualised on a 1.2 % agarose gel. All amplified PCR products were sequenced to confirm the identity of the amplified product.

**Table 1:** Sequence of primers used in this study


Genes	Primer (5ˊ-3ˊ)	Annealingtemperatue (C)	Accession no.	Product size(bp)	Refrence

*TLR1*	F: GGGTCAGCTGGACTTCAGA	63	Gene Bank: U88540.1	250	43
	R: AAAATCCAAATGCAGGAACG				
*TLR2*	F: TCGGAGTTCTCCCAGTTCTCT	60	Gene Bank: NM_003264.3	175	43
	R: TCCAGTGCTTCAACCCACAA				
*TLR3*	F: GTATTGCCTGGTTTGTTAATTGG	60	Gene Bank:NM_003265.2	156	43
	R: AAGAGTTCAAAGGGGGCACT				
*TLR4*	F: TGATGTCTGCCTCGCGCCTG	60	Gene Bank:NM-138554.3	98	32
	R: AACCACCTCCACGCAGGGCT				
*TLR5*	F: CACCAAACCAGGGATGCTAT	60	Gene Bank: NM_003268.5	111	43
	R: CCTGTGTATTGATGGGCAAA				
*TLR6*	F: GCCACCATGCTGGTGTTGGCT	60	Gene Bank: NM-006068.4	101	43
	R: CGCCGAGTCTGGGTCCACTG				
*TLR7*	F: CCTTGAGGCCAACAACATCT	63	Gene Bank: NM_016562.3	285	43
	R: GTAGGGACGGCTGTGACATT				
*TLR8*	F: CTTCGATACCTAAACCTCTCTAGCAC	60	Gene Bank: NM_138636.4	90	43
						R: AAGATCCAGCACCTTCAGATGA
*TLR9*	F: TTCCCTGTAGCTGCTGTCC	60	Gene Bank: NM_017442.3	207	43	
	R: ACAGCCAGTTGCAGTTCACC				
*TLR10*	F: TGCCCACCACAATCTCTTCCATGA	60	Gene Bank: NM-030956.3	184	43
	R: AGCAGCTCGAAGGTTTGCCCA				
*β-actin*	F: CAAGATCATTGCTCCTCCTG	60	Gene Bank: NM-001101	90	43
	R: ATCCACATCTGCTGGAAGG				
*GAPDH*	F: CTCATTTCCTGGTATGACAACGA	60	Gene Bank: NM_002046.4	122	43
	R: CTTCCTCTTGTGCTCTTGCT				


*TLR*; Toll like receptor.

### Cell culture in the presence of sex hormones

To investigate the effect of estradiol and progesterone on *TLR* expression in the OE-E6/E7 cell line, OE-E6/E7 cells were cultured again in triplicates at 37˚C in DMEM (F12) culture medium and water soluble estradiol and progesterone (Sigma-Aldrich, UK) to reach the final concentrations of 0.1, 1, 10, 100 nM and 1, 10, 100, 1000 nM for estradiol and progesterone respectively. In addition, the following combinations of these two (final concentrations) were used in four groups of: control (C, without any additional treatment of sex hormones), menstruation (M, 1 nM progesterone and 0.1 nM estradiol), pre-ovulation (P, 6.5 nM progesterone and 1.5 nM estradiol) and window of implantation (W, 35 nM progesterone and 1 nM estradiol) in 5% CO_2_ atmosphere in 75 ml flasks for 24 hours in the absence of phenol red and serum. In the next step, the effect of sex hormone antagonists on *TLR* expression was evaluated. To do this, OE-E6/E7 cells were divided in two groups. One group was pre-treated for 2 hours with 1 μM ICI 182, 780 (fulvestrant, an estradiol antagonist, Sigma-Aldrich, UK) and the other pre-treated for 2 hours with 0.1 μM RU486 (mifepristone, a progesterone antagonist, Tocris, USA). After pre-treatment, both groups were treated again separately with a combination of estradiol and progesterone based on the four treatment groups (C, M, P and W) in 5% CO_2_ atmosphere in 75 ml flasks for 24 hours in the absence of phenol red and serum.

Cells were next treated for RNA isolation and cDNA synthesis following the same protocol mentioned above. qRT-PCR was performed using the cDNA prepared from the estradiol and progesterone treatment experiments, same primers as in table 1 and SYBR Green Jump Start (Sigma, UK) master mix (containing 10 μl SYBR Green, 7 μl Water, 1 μl of each primer and 1 μl cDNA). The PCR amplification was performed under the following conditions: 50 cycles of 95˚C for 30 seconds, 59˚C to 63˚C for 30 seconds and 72˚C for 30 seconds. All experiments included RT controls and negative controls (no cDNA). qRT-PCR was performed on a Mx3005P QPCR machine (Stratagene, Germany) and results were analyzed using MxPro QPCR software version 4.01. In preliminary experiments, the efficiency of the primer sets of each Q-PCR reaction was established. Variation in *Beta-actin* and *GAPDH* expression as two housekeeping genes was also tested.

The qRT-PCR data were analyzed using the comparative CT method (51). The fold change was calculated as FC=2^-ΔΔCT^.

The results were expressed as mean ± SEM. Significance testing was performed by one-way ANOVA with Tukey’s multiple comparison test. P<0.05 was considered significant.

## Results

### Reverse transcriptase-polymerase chain reaction

**Fig.1:**
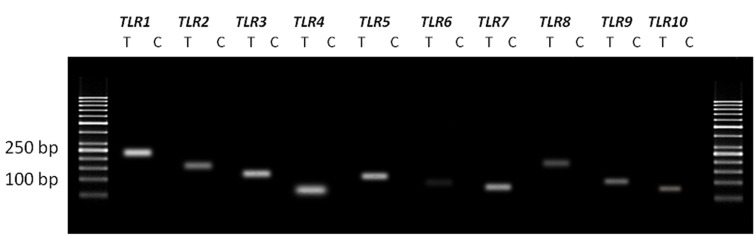
The expression of *TLR1-10* genes in the human fallopian tube tissue. Each pair of primers produced a specific product with the specific predicted size observed in the test (T) samples. C; Control samples (samples without using cDNA) and *TLR*; *Toll like receptor*.

**Fig.2:**
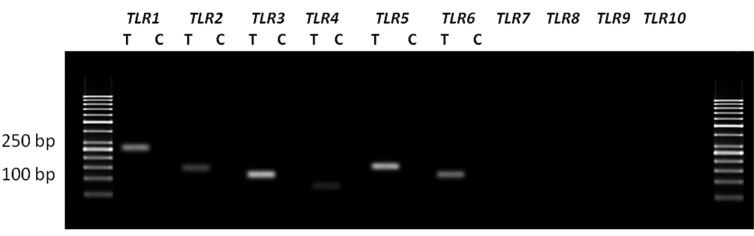
The expression of *TLR1-6* genes in the human fallopian tube cell line (OE-E6/E7). Each pair of primers produced a specific product with the specific predicted size observed in the test (T) samples. C; Control samples (samples without using cDNA) and *TLR*; *Toll like receptor*.

Figure 1 shows the results of RT-PCR of *TLR1- 10* genes in the human fallopian tube tissue. Figure 2 shows the results of RT-PCR of *TLR1-6* genes in OE-E6/E7 cells. Size of all amplified PCR products were as predicted and sequencing verified correct amplification of each gene. No product was amplified in negative control samples, indicating absence of genomic DNA contamination.

### Immunostaining

**Fig.3:**
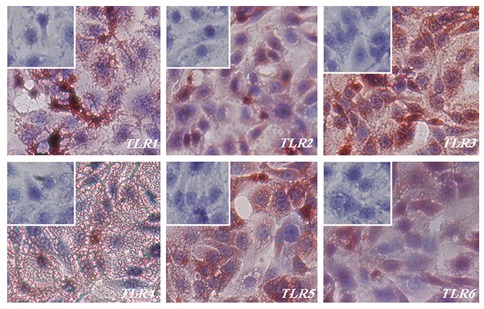
Immunohistochemical staining of *TLR1-6* in the OE-E6/E7 cell line. Positive staining is red, negative staining is blue. Small inserts show blocking of anti *TLR1-6* antibodies with respective specific peptides. *TLR*; *Toll like receptor*.

Formalin-fixed slides were used to study the distribution of *TLR1-6* in OE-E6/E7 cells. Positive immunostaining for all six *TLRs* was observed with *TLR1*, 2, 4 and *TLR6* displaying moderate staining in this epithelial cell line. However, strong staining for *TLR3* and *TLR5* was observed. The immunocytochemical localisation of *TLR1-6* is shown in figure 3.

### Quantitative polymerase chain reaction

**Fig.4:**
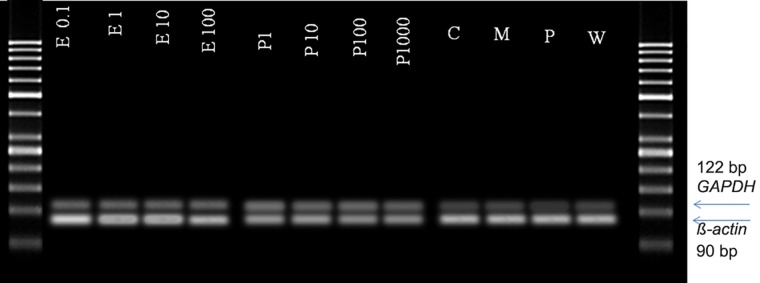
The stable expression of GAPDH and β-actin in OE-E6/E7 under variable hormonal treatments. E; Estradiol, P; Progestrone, C; Control, M; Menstruation and W; Window of implantation.

**Fig.5:**
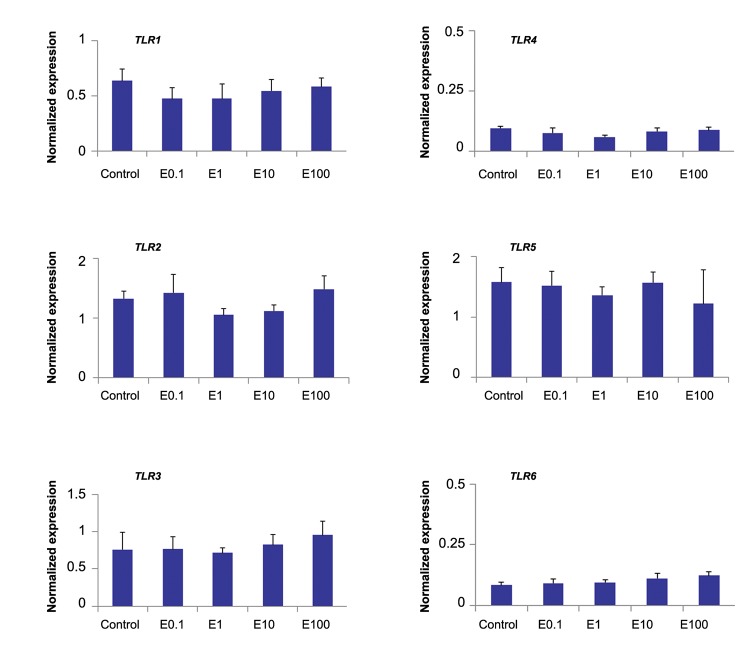
Expression of *TLR1-6* in OE-E6/E7cultured with estradiol. Mean ± SEM of normalized expression values (with β-actin) for *TLR1-6* genes in OE-E6/E7 cultured with different concentrations of estradiol (control: 0 nM, E 0.1: 0.1 nM, E1: 1 nM, E10: 10 nM and E100: 100 nM or 1 μM). No significant results were obtained. *TLR*; *Toll like receptor*.

**Fig.6:**
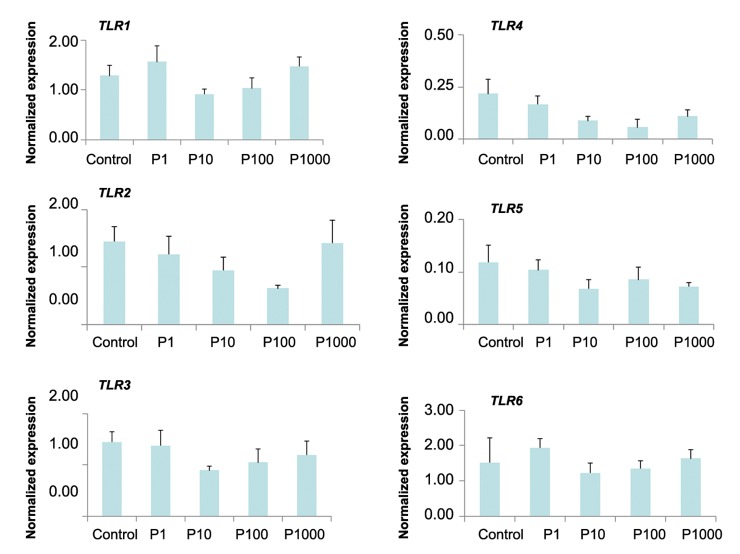
Expression of *TLR1-6* in OE-E6/E7cultured with progesterone. Mean ± SEM of normalized expression values (with β-actin) for *TLR1- 6* genes in OE-E6/E7 cultured with different concentrations of progesterone (control: 0 nM, P1: 1 nM, P10: 10 nM, P100: 100 nM and P1000: 1000 nM or 1 μM). No significant results were obtained. *TLR*; *Toll like receptor*.

**Fig.7:**
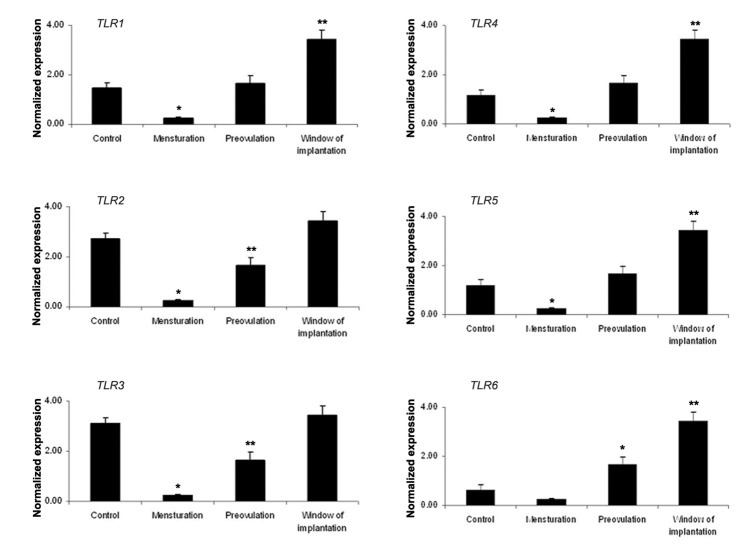
Expression of *TLR1-6* in OE-E6/E7 cultured with a combination of estradiol and progesterone concentrations. Mean ± SEM of normalized expression values (with β-actin) for *TLR1-6* genes in OE-E6/E7 cultured with a combination of estradiol and progesterone. Control (C, without any additional treatment of sex hormone), menstruation (M, 1 nM progesterone and 0.1 nM estradiol), pre-ovulation (P, 6.5 nM progesterone and1.5 nM estradiol) and window of implantation (W, 35 nMprogesterone and 1 nM estradiol) (control: 0 nM, P1: 1 nM, P10: 10 nM, P100: 100 nM and P1000: 1000 nM or 1 μM). Star denotes statistically significant differences. *TLR*; *Toll like receptor*.

A stable expression of *Human ß-actin* and *GAPDH* genes with variable hormonal treatments was observed (Fig .4).The quantitative expression profiles of *TLR1-6* genes in OE-E6/E7 cells treated with varying concentrations of estradiol and progesterone are shown in figures 5 and 6 respectively. The relative expression of *TLR1-6* genes did not significantly differ in response to different concentrations of either estradiol or progesterone. The mean relative expression of *TLR1-6* differed considerably between P, M, W and C groups (Fig .7). The Pattern of *TLR* expression in P, M and W were significantly different among P, M and W groups. Also, by using 1 μM of fulvestrant (estradiol antagonist) or 0.1 μM mifepristone (progesterone antagonist) 2 hours prior to the combined estradiol and progesterone treatment, the mean relative expression of *TLR1-6* did not differ markedly in C, P, M and W groups (Figes.8, 9).

**Fig.8:**
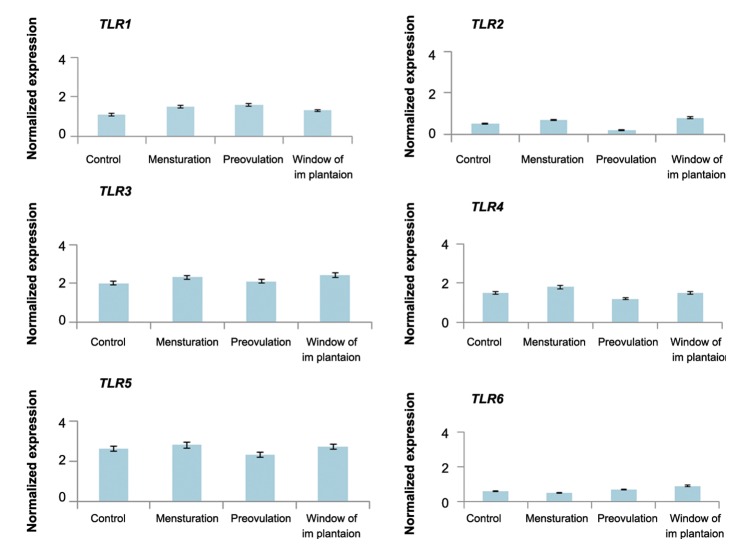
Expression of *TLR1-6* in OE-E6/E7 precultured with fulvestrant, then cultured with a combination of estradiol and progesterone concentrations. Mean ± SEM of normalized expression values (with β-actin) for *TLR1-6* genes in OE-E6/E7 pre-cultured with fulvestrant for 2 hours, then cultured with a combination of estradiol and progesterone in 4 groups (C, M, P, W). No significant results were obtained. *TLR*; *Toll like receptor*.

**Fig.9:**
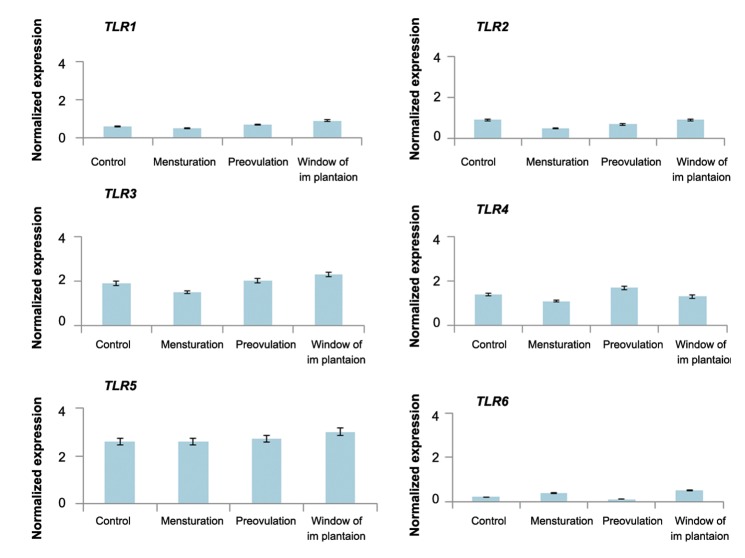
Expression of *TLR1-6* in OE-E6/E7 pre-cultured with mifepristone and cultured with a combination of estradiol and progesterone in 4 groups (C, M, P, W). Mean ± SEM of normalized expression values (with β-actin) for *TLR1-6* genes. No significant results were obtained. *TLR*; *Toll like receptor*.

## Discussion

Epithelial cells are the first layer of defense against pathogens ascending FRT. Considering their role in protecting the tract from the external environment, *TLRs* are expected to be present in this tissue. Several studies have investigated the presence and the role of *TLRs* in FRT especially within the fallopian tube (34,42,52). Previously, Pioli et al. (39), showed the presence of *TLR1-6* in the FRT as well as the accessory molecule CD14 and the molecular adapter MyD88 both of which are needed for *TLR* signalling. They also observed expression of *TLR1-6* at the transcript level in fallopian tubes. In agreement with this report, we previously showed the localization of *TLR1-6* in the fallopian tube using immunohistochemistry (36). Both observations are in agreement with our findings in this study that demonstrate the presence of *TLR1-6* transcripts in human fallopian tube tissues and cell line. The expression of *TLR4* protein and transcript has been detected in human endometrial epithelial cells, stromal cells (38) and fallopian tube stromal fibroblasts (53). However, *TLR4* expression was not detected in fallopian tube epithelial cells by Itoh et al. (53). In another study Hart et al. (42), reported the expression of *TLR7–9* in the fallopian tube, uterine, endometrium, cervix and ectocervix, while *TLR10* expression was restricted to the fallopian tube. 

In this study, although expression of *TLR1-10* was detected in human fallopian tube tissues, expression of only *TLR1-6* was detected in OE-E6/ E7 cells. This may be due to difference in features of the fallopian tube tissue and this specific cell line. Fallopian tube tissue contains an epithelial layer, stroma and capillaries supplying blood. Within the epithelial layer of the fallopian tube tissue, some of the cells are ciliated and some are known as secretory cells. The OE-E6/E7 cell line is only an isolated fallopian tube epithelial layer cell line. Therefore, the expression of *TLR* genes and their level of expression in this cell line is likely to be different compared with the original tissue consisting of different types of cells. The other potential explanation for the absence of expression of some *TLRs* in OE-E6/E7 cells may be due to differences between immortalised cell lines and their original parental primary cells. It is known that cell lines may undergo changes due to the process of immortalization. However, the characteristics of this immortalized cell line have been compared to the parental human fallopian tube epithelium in several investigations. For example, human oviduct-specific glycoprotein, estradiol receptors and cytokeratin molecules have been found to be produced in both primary fallopian tube cells and OE-E6/E7 cells (50,54,55). 

The FRT environment is under the control of sex hormones during the menstrual cycle. Sex hormones not only regulate anatomical and histological characteristics of this tract (56,57), they are also involved in the influx and localization of immune cells in this tract (58,60). For example, uterine natural killer (uNK) cells are found in the human uterus in large numbers and are spread throughout the endometrium with increasing numbers as the menstrual cycle progresses (61,62). In addition, the adaptive immune system is also influenced by the changing levels of sex hormones during the menstrual cycle. Antigen presentation has been shown to be suppressed in response to increasing concentrations of estradiol (63,64). Sex hormones including estradiol and progesterone tightly control the distribution of macrophages within the endometrium (65,68). Another study demonstrated that estradiol, which is secreted by the ovary during the menstrual cycle, modulates epithelial cells and other immune cells in FRT directly and indirectly to regulate a wide range of immune functions specific to each site in FRT (69). Nasu and Narahara (49) also showed that antigen-presenting cells in the uterus and vagina are responsive to estradiol where antigen presentation as well as co-stimulatory molecule expression is inhibited by estradiol. Furthermore, they suggested that antigen-presenting cells in the uterus and vagina respond to selected *TLR* agonists with altered antigen presentation. 

It is therefore likely that the action of *TLRs* is also modified by changing concentrations of estradiol and progesterone. Although all *TLR* molecules are expressed throughout the cycle, the majority of these genes are expressed at their lowest level during menstrual and proliferative stages of the cycle (34). At follicular stage of the cycle, while progesterone levels are at their lowest, estradiol levels are at their highest. This may indicate an inhibitory effect of estradiol and/or enhancing influence of progesterone on the expression of *TLR* molecules in FRT, especially in the endometrium. It is plausible that this alteration in *TLR* gene expression may even influence the function of *TLRs* in mediating innate immune responses in FRT. To test these hypotheses, we examined the effect of sex hormones on *TLR* expression in the OE-E6/ E7 cell line. We used various concentrations of estradiol and progesterone to determine the effects of these sex hormones on *TLR* expression. No significant alteration in the relative expression of *TLR1-6* transcripts was observed in the fallopian tube cell line in response to different concentrations of estradiol and progesterone. These results agreed with the findings of Lesmeister et al. (47) who also showed that *in vitro* treatment of endometrial epithelial cell line (RL95-2) with 17betaestradiol did not have an effect on *TLR3* transcript or protein expression. Also, in a recent study on human uterine epithelial cells and the ECC-1 uterine epithelial cell line, Nasu and Narahara (49) demonstrated that estradiol either alone or prior to treatment with poly (I:C), had no effect on the expression of interferon β (IFNβ) or interferonstimulated genes (ISG). However, another study showed that production and secretion of protective antimicrobials including human β defensin-2 (HBD2) and secretory leukocyte protease inhibitor (SLPI) are directly upregulated by estradiol. On the contrary, estradiol inhibited LPS and poly (I:C)induced secretion of macrophage inhibitory factor (MIF), interleukin 6 (IL-6) and IL-8 in primary uterine epithelial cells (70). 

In the next step of our study, combined effect of estradiol and progesterone was evaluated. Expression of *TLR1-6* was higher when the level of sex hormones in culture media was comparable to their concentration in serum during the window of implantation (group W) in the menstrual cycle. These results agreed with our previous findings where higher expression of *TLRs* was observed during the secretory phase in human endometrium (34,71). These findings demonstrated a stimulatory effect of co-presence of both sex hormones (M, P and W) on *TLR1-6* expression. 

A recent study reported the fluctuation of *TLR* responsiveness in peripheral blood throughout the menstrual cycle (72). In agreement with our data, during the follicular phase, lower levels of IL-6 and tumor necrosis factor (TNF)-α following stimulation with the *TLR2* agonist, lower levels of IL-1β, IL-6 and TNF-α following stimulation with the *TLR4* agonist LPS, and lower levels of IL-1β and TNF-α following stimulation of whole blood with the *TLR5* agonist flagellin was observed when compared with the early luteal phase (72). Jorgenson et al. (46) also illustrated that *TLR3* transcript level in primary endometrial epithelial cells is menstural cycle-dependent. They showed *TLR3* was expressed throughout the menstrual cycle but at its highest during the secretory phase of the cycle. In another study, Yao et al. (73) reported that except *TLR11*, the expression of *TLR1-10* is cycle-dependent in mouse. Whether the above mentioned findings are due to estradiol, progesterone or their combined effect on FRT epithelium remains to be deciphered. 

When fulvestrant (estradiol antagonist) or mifepristone (progesterone antagonist) was used, the combined effect of estradiol and progesterone on *TLR* expression was inhibited. These data thus confirm our results that on the expression of *TLR16* is not affected by progesterone and estradiol individually *TLR1-6* but *TLR1-6* under the synergistic/additive effect of the combination of estradiol and progesterone. 

In performing this investigation, we took several precautionary measures. The effect of hormones in cell culture experiments was tested in the absence of phenol red and serum. Phenol red has estradiolic properties and serum may contain small molecules with estradiolic effects and if present, may hamper the results of experiments. 

In *in vitro* culture systems and particularly in the presence of blood or serum samples, progesterone degrades quickly (74). To avoid early degradation of hormones during our cell culture experiments, we used water soluble estradiol and progesterone (Cyclodextrin-encapsulated 17β-estradiol and progesterone) which are stable and tested for cell culture applications. These compounds have been used in several similar investigations (75,79). Furthermore, our results clearly demonstrated that these compounds do not alter *TLR* gene expression in cultured cells. 

It seems that the pattern of *TLR* expression under different concentrations of estradiol and progesterone (mimicking those of the menstrual cycle) is similar to the pattern of *TLR* expression in endometrial tissue during the menstrual cycle (34). On the other hand, higher expression of *TLRs* in the proliferative phase compared with menstruation as well as their higher expression in the secretory phase than other phases show that safety in the human fallopian tube at the time of ovulation or early embryo development is a key factor. 

Future studies should be directed towards understanding the role of signalling pathways that enable estradiol and progesterone to modulate the expression and function of *TLRs* in FRT. 

## Conclusion

This study firmly points to the involvement of sex hormones in modulation of *TLR* gene expression in human fallopian tube cells. Further experiments should be undertaken to reveal the regulatory mechanism(s) and signalling pathway(s) responsible for the effect of sex hormones in modulating innate immunity in human FRT. 
